# AE37 HER2-targeted vaccine in the prevention of breast cancer recurrence: A mini narrative review of current evidence

**DOI:** 10.1097/MD.0000000000036793

**Published:** 2023-12-29

**Authors:** Gbolahan Olatunji, Emmanuel Kokori, Ismaila Ajayi Yusuf, Olumide Akinmoju, Timilehin Isarinade, Rosemary Komolafe, Aminat Akinoso, Adeola Akinboade, Osadebamwen Osaghae, Muili Abdulbasit, Nicholas Aderinto

**Affiliations:** a Faculty of Clinical Sciences, University of Ilorin, Ilorin, Nigeria; b Department of Medicine and Surgery, Obafemi Awolowo University Teaching Hospital, Ife, Nigeria; c Department of Medicine and Surgery, University of Ibadan, Ibadan, Nigeria; d Johns Hopkins Bloomberg School of Public Health, Washington, DC; e Department of Medicine and Surgery, Ladoke Akintola University of Technology Teaching Hospital, Ogbomoso, Nigeria.

**Keywords:** AE37 HER2, breast cancer, prevention, vaccine

## Abstract

Breast cancer remains a significant global health challenge, necessitating innovative therapeutic strategies. This review synthesizes findings from multiple studies investigating the safety profile and efficacy of the AE37 human epidermal growth factor receptor 2 (HER2)-targeted vaccine, offering insights into its potential role in breast cancer immunotherapy. A systematic search of electronic databases, including PubMed, MEDLINE, Scopus, and Web of Science, was conducted to identify relevant articles published up to October 2023. The search strategy utilized a combination of keywords, including “AE37 HER2 vaccine,” “breast cancer recurrence prevention,” and related terms. Boolean operators (AND, OR) were employed to refine the search. The AE37 vaccine exhibited a favorable safety profile across all studies, with minimal adverse effects reported. Efficacy outcomes varied, with promising trends observed in specific breast cancer subgroups, such as advanced-stage, HER2 under-expressed, and triple-negative breast cancer patients. Subgroup analyses suggested potential benefits, emphasizing the need for precise patient stratification. While the AE37 HER2-targeted vaccine demonstrates a promising safety profile and potential efficacy in specific breast cancer subgroups, an understanding requires addressing identified limitations and advancing research in nuanced directions. This paper provides a foundation for navigating the complex landscape of breast cancer immunotherapy with the AE37 vaccine.

## 1. Introduction

Breast cancer, a pervasive global health challenge, registers a staggering 2.3 million new cases annually, making it a significant contributor to female morbidity and mortality.^[1]^ Comprising 23% of all female malignancies, it stands responsible for 14% of cancer-related deaths.^[2]^ However, the gravity of the situation extends beyond the initial diagnosis and treatment phase, with breast cancer recurrence proving to be a formidable adversary. Loco-regional recurrences afflict 8% to 10% of women initially diagnosed with breast cancer, while an even more disheartening 15% to 30% confront the grim specter of distant metastases.^[3]^

The complexity of breast cancer recurrence demands a distinct and dedicated approach to its management. Notably, estrogen receptor (ER)-negative breast cancers within the first 5 years post-diagnosis exhibit heightened susceptibility compared to their ER-positive counterparts, with risks persisting and escalating over a decade.^[4]^ Human epidermal growth factor receptor 2 (HER2) status has emerged as a pivotal determinant. The 4 major molecular subtypes of breast cancer, categorized by the expression of hormones and HER2 receptors, include luminal A, luminal B, HER2-enriched, and basal-like triple-negative. HER2-enriched cancers, along with a subset of luminal B breast cancers, feature HER2 overexpression.^[5]^

Within this challenging context, the AE37 HER2-targeted vaccine represents a promising Frontier for innovative solutions. Human epidermal growth factor receptor 2 (HER2), a key protagonist in breast cancer development, plays a pivotal role, particularly in HER2-positive breast cancer—a malignancy afflicting 20% to 30% of breast cancer cases.^[6]^ Known for their aggressive nature and grim prognostic outlook, HER2-positive breast cancers pose a considerable threat, with 20% to 30% of patients facing recurrence.^[7]^

The AE37 HER2-targeted vaccine, a groundbreaking peptide-based immunotherapy, emerges as a beacon of hope in breast cancer treatment and prevention. At the heart of AE37’s potential lies its mechanism of action, initiated upon uptake by antigen-presenting cells. Subsequently, CD8 + T-cells, orchestrated by the presence of the peptide antigen, generate specific cytotoxic lymphocytes that identify and dismantle tumor cells expressing the antigen.^[8]^ In contrast to other peptide vaccines, such as E75, which functions by instigating CD8 + T-cells to obliterate tumor cells directly, AE37 adopts a revolutionary approach. It harnesses the power of an major histocompatibility complex (MHC) class II peptide, stimulating CD4 + helper T cells and thereby elevating the immune response. Despite MHC class II peptides having lower binding affinity than class I, AE37 ingeniously leverages its hybrid nature by covalently linking the li-key peptide to the AE36 amino terminus, effectively enhancing antigen presentation.^[9,10]^ AE37’s prowess in binding MHC class II is amplified a staggering 250-fold when contrasted with AE36, another HER2 vaccine.^[8]^ The advantages of peptide-based cancer vaccines, including ease of synthesis, cost-effectiveness, and tolerable side effects, further position AE37 as a promising avenue for exploration.^[5]^ Despite not directly targeting the HER2 protein, the AE37 vaccine acts by directly activating CD4 + lymphocytes, exposing the immune system to the HER2 protein, which is then recognized as foreign and set up for destruction.^[11,12]^

**Figure 1.**
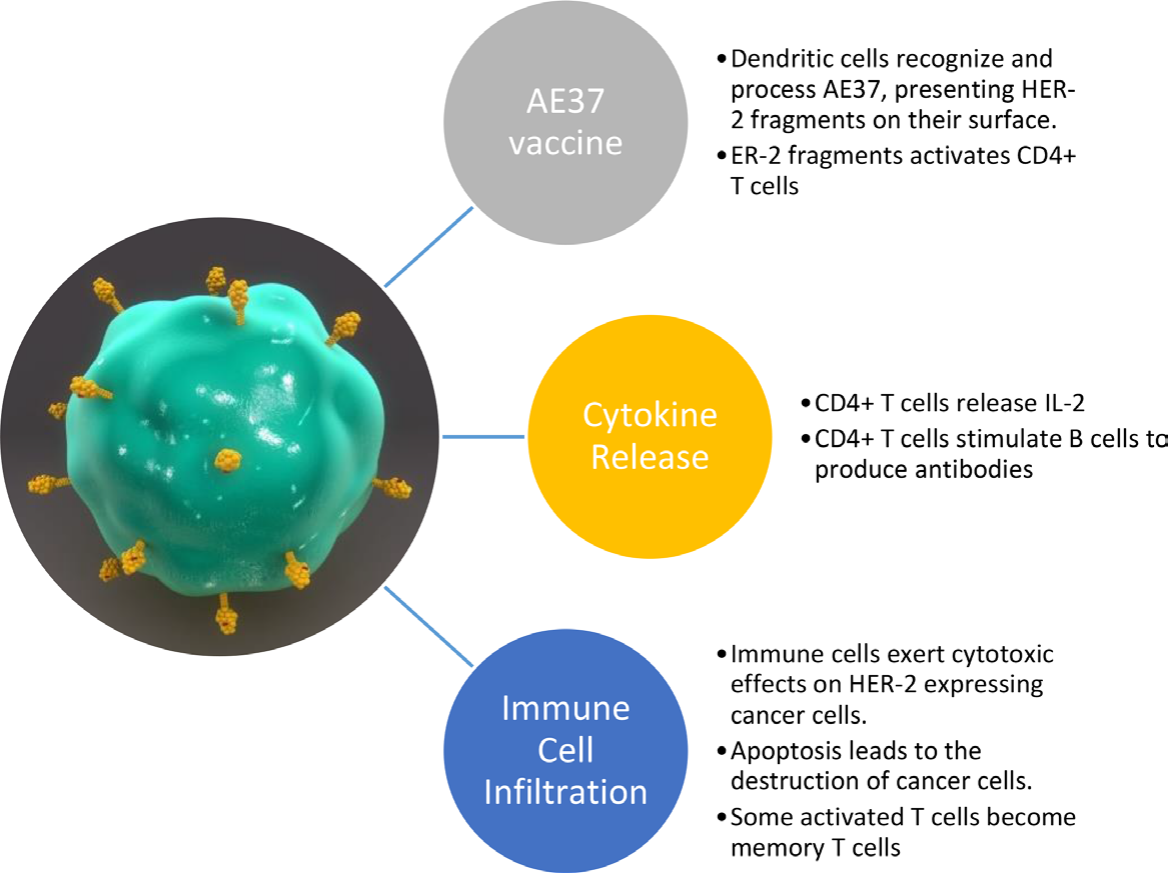
Anticancer molecular mechanisms of AE37 HER-2 targeted vaccine.

The immune response elicited by the AE37 vaccine, as evidenced in a 2014 study, demonstrates participants expressing antibodies to the HER2 protein. See Figure 1. As a peptide-based cancer vaccine stimulating CD4 + T helper cells, AE37 exhibits potential in inducing memory cells in the immune system alongside CD8 + cytotoxic T cells, contributing to an effective immune response.^[13,14]^ These T cells are essential for the body’s adaptive immune response to foreign bodies, pathogens, and tumors, holding promise in sometimes averting recurrence.^[15]^ This review explores the current evidence on the AE37 HER2-targeted vaccine in treating breast cancer recurrence.

## 2. Methodology

A systematic search of electronic databases, including PubMed, MEDLINE, Scopus, and Web of Science, was conducted to identify relevant articles published up to October 2023. The search strategy utilized a combination of keywords, including “AE37 HER2 vaccine,” “breast cancer recurrence prevention,” and related terms. Boolean operators (AND, OR) were employed to refine the search.

### 2.1. Selection criteria

Clinical trials focused on the AE37 HER2-targeted vaccine, breast cancer recurrence prevention, and related topics. No time limit were used for the study search. Studies that did not meet the criteria, such as those not written in English, not peer-reviewed, or irrelevant to the specified focus, were excluded. Titles and abstracts of identified articles were screened to assess their relevance to the review objectives. Full-text articles were selected for further evaluation based on the inclusion criteria. A consensus-based approach was adopted in cases of disagreement between reviewers. Data were extracted from selected articles. A standardized data extraction form was utilized to ensure consistency.

The extracted data were synthesized and organized to develop a coherent narrative. Study design, patient characteristics, and key findings grouped studies. Patterns, trends, and inconsistencies in the literature were identified to provide an overview. A thematic analysis was conducted to identify common themes and variations across studies. Findings were categorized based on efficacy, safety, and integration of the AE37 vaccine into breast cancer recurrence treatment strategies.

## 3. Results

This review synthesizes findings from studies investigating HER2-targeted vaccines for breast cancer recurrence prevention. See Table 1. The studies, primarily clinical trials, collectively enrolled 48 to 456 participants, offering a broad representation of breast cancer populations. The focus was on disease-free individuals, emphasizing node-positive or high-risk node-negative patients. Treatment protocols spanned initial inoculations with subsequent booster vaccinations, demonstrating variability in dosing and intervals. Notably, dosages included specific quantities of the peptide, granulocyte-macrophage colony-stimulating factor (GM-CSF), or a combination of both. The trials evaluated disease-free survival rates as the primary efficacy outcome, with findings varying across treatment arms and subgroups. Subgroup analyses yielded noteworthy insights, indicating potential benefits in advanced-stage patients, those with HER2 under-expression, and those with triple-negative breast cancer. Despite overall safe profiles, observed toxicities during the vaccination series were generally mild, primarily graded as 1 or 2.

**Table 1:** Study characteristics.

Author/year	Study design	Sample size	Study population	Duration of treatment and dosing	Safety outcomes	Efficacy outcomes	Key conclusions
Brown et al (2020)	Clinical Trial, Phase II	456 patients	In this 4-arm, prospective, randomized, single-blinded, multi-center phase II trial, disease-free node positive and high-risk node negative breast cancer patients enrolled after standard of care therapy	Six inoculations were given in 3–4 week intervals administered intradermally consistently in the same lymph node distribution (same arm or thigh) in each patient. Patients in each treatment arm received 500 mcg of the peptide and 125 mcg of GM-CSF, while the control arm received 125 mcg of GM-CSF alone. After the initial 6 inoculations, patients were given a total of 4 booster inoculations at 6-month intervals beginning 1 year after each subject’s date of enrollment		The AE37 arm had no difference in DFS as compared to CG, but prespecified exploratory subgroup analyses showed a trend towards benefit in advanced stage (*P* = .132, HR 0.573 CI 0.275–1.193), HER2 under-expression (*P* = .181, HR 0.756 CI 0.499–1.145), and triple-negative breast cancer (*P* = .266, HR 0.443 CI 0.114–1.717). In patients with both HER2 under-expression and advanced stage, there was significant benefit in the VG (*P* = .039, HR 0.375 CI 0.142–0.988) as compared to CG. The GP2 arm had no significant difference in DFS as compared to CG, but on subgroup analysis, HER2 positive patients had no recurrences with a trend toward improved DFS (*P* = .052) in VG as compared to CG.	This phase II trial reveals that AE37 and GP2 are safe and possibly associated with improved clinical outcomes of DFS in certain subgroups of breast cancer patients. With these findings, further evaluations are warranted of AE37 and GP2 vaccines given in combination and/or separately for specific subsets of breast cancer patients based on their disease biology.
Mittendorf et al (2016)	Clinical Trial, Phase II	180	The trial enrolled 180 HLA-A2 + patients; 89 were randomized to the GP2 + GM-CSF vaccine group and 91 to the GM-CSF only control arm (figure)		For patients receiving GP2 + GM-CSF, maximum local toxicities experienced during the primary vaccination series (PVS) were grade 1 (70%), grade 2 (28%), or grade 3 (1%). The most common toxicities included erythema, induration and pruritis; the grade 3 toxicity was induration.	The Kaplan–Meier estimated 5-year DFS rate in the ITT analyses was 88% (95% CI: 78–94%) in vaccinated vs 81% (95% CI: 69–89%) (*P* = .43) in control patients after a 34 month median follow-up. In the per-treatment analysis, the estimated 5-year DFS rates were 94% (95% CI: 83–98%) and 85% (73–92%) (*P* = .17). In IHC 3+/FISH + patients, the estimated 5-year DFS rate was 94% (82–98%) in vaccinated patients (n = 51) vs. 89% (71–96%) in control patients (n = 50), (*P* = .86) in the ITT analyses and 100% vs. 89% (71–96%) in vaccinated vs. control patients in the per-treatment analyses (*P* = .08).	While the overall ITT analysis did not demonstrate benefit to vaccination, this trial confirmed that the GP2 vaccine is safe and suggests that vaccination may have clinical activity, particularly in patients with HER2 overexpression who received the full vaccine series (i.e., per-treatment group).
Greene et al (2015)	Phase II trial	391	After completion of standard of care therapy, pts with any level of HER2 expression (IHC1-3+) were randomized to the vaccine group (VG) to receive 6 monthly intradermal inoculations of AE37 + GM-CSF followed by 4 booster vaccinations every 6 months			Of 301 enrolled pts, 154 were randomized to the VG and 147 to the CG. There were no differences in age, grade, receptor status, tumor size or nodal status between groups (all *P* ≥ .1). Five-year DFS rates were not different with 82% (VG) vs 79.9% (CG), HR = 0.96, *P* = .9. There were trends in several subgroups. In HER2 1–2 + pts (77 VG vs 80 CG), 5 yr DFS was 79% (VG) vs 75% (CG), HR 0.76, *P* = .48. In ER/PR− pts (59 VG vs 56 CG), 5 yr DFS was 83% (VG) vs 77% (CG), HR 0.67, *P* = .36. In TNBC pts (25 VG vs 26 CG), 5 yr DFS was 67% (VG) vs 62% (CG), HR 0.65, *P* = .42.	Overall, the final timed analysis of this phase II trial shows no statistical differences between treatment arms; however, it does identify particular pt populations where the vaccine may have efficacy. The ER/PR negative and triple negative pts appear to derive greatest benefit from AE37 vaccination with reductions in relative risk of recurrence of 33% and 35%, respectively.
Anastasopoulou et al (2016)	Phase II Trial	48	The trial is enrolling NP or high-risk NN patients with any degree of HER2 expression (IHC 1–3 + or FISH > 1.2) rendered disease-free following standard of care therapy.	The vaccine group (VG) received AE37 + GM-CSF and control group (CG) GM-CSF alone in 6 monthly i.d. inoculations followed by boosters administered every 6 months × 4.		Mean dermal reactions (orthogonal mean in mm) in vaccinated patients was 25.9 ± 3.13 at completion of the PVS (R6) and increased to 35.47 ± 4.35 at BRC24 (*P* = .01). VG patients increased their proliferation response (stimulation index, SI) to AE36 from 0.97 ± 0.046 at baseline (R0) before vaccination to 2.27 ± 0.57 at R6 (*P* = .0003) which was maintained until BRC24 (SI 2.21 ± 0,33, *P* < .0001). The number of IFN-γ specific spots/106 PBMC increased from 26.88 ± 12.36 at R0 to 40.35 ± 17.02 (*P* = .07) at R6, up to 62 ± 16.82 (*P* = .0076) at BRC24.	Our data demonstrate that AE37 vaccine boosters enhance the immune responses against HER elicited during the PVS, thus sustaining long lasting immunity, a prerequisite for possible clinical efficacy which is currently being evaluated
Erika J. Schneble (2013)	Phase II Trial	208	After completing standard of care therapy, high-risk, disease-free breast cancer pts with HER2 LE (IHC 1+, 2+) or OE (IHC 3+) tumors are eligible for enrollment in our phase II trial evaluating the HER2-derived peptide vaccines, AE37 (MHC Class II, HLA-nonrestricted epitope) and GP2 (MHC Class I, HLA-A2 + restricted epitope).	Median follow up of 39 months		88 HLA-A2 + (48 OE and 40 LE) and 115 HLA-A2− (48 OE and 67 LE). A2 + and A2- OE pts exhibited significant difference in respect to age (52 vs. 47, *P* = .013) and tumor size (41.7% vs. 68.1% ≥ 2 cm, *P* = .01) respectively. With median follow-up of 39 months, recurrence rates were again compared between A2 + vs A2- pts (11.4% vs. 11.3%, *P* = .50) as well as HER2 subgroups (OE, 6.25% vs. LE, 15.8%, *P* = .02). The latter difference was particularly prominent among the A2− pts (A2 −/OE, 4.2% vs 16.4%, *P* = .03) but less so in the A2+ pts (A2+/OE, 8.3% vs. A2+/LE, 15%, *P* = .26). With no recurrence difference between LE A2 subgroups (LE/A2+, 15% vs. LE/A2−, 16.4%, *P* = .54), the OE subgroup comparison was notable for 50% increased disease recurrence in A2 + pts (A2+/OE, 8.3% vs. A2 −/OE, 4.2%, *P* = .33).	While there is no difference in recurrence between A2+ and A2− pts overall or among the HER2 LE subset, there does appear to be a difference in the HER2 OE subset. Compared to A2+/OE pts, the A2−/OE pts exhibit 50% decreased disease recurrence despite significantly younger age and larger tumor size. These findings need to be confirmed but are important given that many peptide vaccines target A2+ pts. Therefore, these results may impact future trial design.

### 3.1. Safety profile of AE37 HER2-targeted vaccine

Consistent evidence indicates a favorable safety profile and well-tolerated nature of the vaccine. The study by Brown et al (2020), involving 456 patients, demonstrated that the AE37 arm exhibited a safety profile comparable to the control arm.^[11]^ No significant differences in adverse effects were noted between the 2 groups. Mittendorf et al (2016), in their Phase II trial with 180 HLA-A2 + patients, reported a favorable safety profile for the GP2 + GM-CSF vaccine. Maximum local toxicities during the primary vaccination series were mostly grade 1 or 2, with rare occurrences of grade 3 toxicities.^[9]^ This suggests that the vaccine was well-tolerated in the patient population. Similarly, Greene et al (2015), in their Phase II trial with 391 participants, found no statistically significant differences in adverse effects between the AE37 + GM-CSF vaccination group and the control group.^[16]^ The comparable safety outcomes indicate the well-tolerated nature of the AE37 + GM-CSF vaccine.

Anastasopoulou et al (2016), involving 48 patients, demonstrated that AE37 vaccine boosters were well-tolerated. Dermal reactions increased during the primary vaccination series, and immune responses against HER were enhanced, indicating a positive safety profile for AE37 vaccine boosters.^[15]^ In the study by Erika J. Schneble et al (2013), which included 208 patients, no specific adverse effects or safety concerns related to the AE37 HER2-derived peptide vaccine were reported.^[17]^ While the primary focus was on disease recurrence, the absence of safety concerns suggests an overall favorable safety profile for AE37 in this trial.

### 3.2. Efficacy of AE37 HER2-targeted vaccine

In Brown et al’s 2020 multicenter trial involving 456 patients, the AE37 arm exhibited comparable disease-free survival (DFS) to the control arm.^[11]^ However, subgroup analyses unveiled potential benefits in advanced-stage, HER2 under-expressed, and triple-negative breast cancer patients. Notably, significant benefits were observed in the vaccine group for patients with both HER2 under-expression and advanced stage, indicating a context-specific advantage. Similarly, Mittendorf et al (2016) trial demonstrated an 88% 5-year DFS rate in the AE37 arm compared to 81% in the control arm.^[9]^ Although the overall intention-to-treat analysis did not show statistical significance, the per-treatment analysis suggested higher DFS rates, particularly in patients with HER2 overexpression. This emphasizes the potential clinical activity of the GP2 vaccine, especially in specific subgroups. In addition, Greene et al’s (2015) study with 391 participants did not reveal statistically significant differences in the overall 5-year DFS rates between the AE37 + GM-CSF vaccination group and the control group.^[16]^ However, subgroup analyses indicated trends, showcasing potential efficacy in patients with ER/progesterone receptor-negative and triple-negative breast cancer. Despite the lack of statistical significance, identifying specific patient populations with potential benefits is crucial for targeted approaches.

Anastasopoulou et al (2016) trial involving 48 patients, AE37 vaccine boosters enhanced immune responses against HER, indicating sustained immunity.^[15]^ While not directly measuring DFS, this finding underscores the potential long-term efficacy of AE37 and ability to bolster immune responses. Erika J. Schneble et al (2013) study with 208 patients, the AE37 HER2-derived peptide vaccine also demonstrated variable recurrence rates between patient subgroups.^[17]^ While there was no significant difference in recurrence between A2+ and A2− patients overall, intriguing variations emerged in the HER2 overexpressed subset. Notably, A2−/OE patients exhibited a 50% decreased disease recurrence despite being younger and having larger tumors. These results suggest the need for further confirmation and highlight the importance of considering patient-specific factors in trial design.

## 4. Discussion

The findings presented in this review of studies on the AE37 HER2-targeted vaccine open avenues for a nuanced discussion regarding its clinical implications, limitations, and prospects for future research.

The consistent demonstration of a favorable safety profile and potential efficacy in specific subgroups suggests that AE37 holds promise as a therapeutic option for breast cancer patients. The observed benefits in advanced-stage, HER2 under-expressed, and triple-negative breast cancer patients, as highlighted in studies by Brown et al (2020) and Greene et al (2015), signify the importance of patient stratification for targeted approaches.^[9,11]^ Moreover, the encouraging outcomes in patients with both HER2 under-expression and advanced stage, as indicated by Brown et al (2020), emphasize the need for a nuanced understanding of the contextual factors influencing vaccine efficacy.^[11]^ The study by Mittendorf et al (2016) contributes to the discussion by indicating potential clinical activity, particularly in patients with HER2 overexpression who received the full vaccine series.^[16]^ This insight directs attention towards the importance of dosing and treatment duration in realizing the clinical benefits of AE37, underscoring the need for personalized treatment strategies.

Furthermore, the sustained immune responses against HER, demonstrated by Anastasopoulou et al (2013), provide a valuable perspective on the long-term efficacy of AE37.^[15]^ While not directly measuring DFS, this finding prompts considerations of the vaccine’s ability to bolster immune memory, which may contribute to prolonged protection against disease recurrence.

Despite the promising aspects, it is imperative to acknowledge the limitations inherent in these studies. The variable recurrence rates observed by Erika J. Schneble et al (2013) and the absence of statistical significance in certain analyses, such as the overall intention-to-treat analysis in Brown et al (2020), warrant caution in interpreting the results.^[11,17]^ Patient heterogeneity, differences in trial designs, and the relatively modest sample sizes in some studies contribute to the complexity of drawing definitive conclusions. Additionally, the focus on HER2 subtypes and the intricate interplay with immunogenetic factors, as seen in the study by Erika J. Schneble et al (2013), highlights the need for a more nuanced understanding of the patient-specific factors influencing vaccine response.^[11]^ The limited representation of certain subgroups in these trials necessitates further investigation to elucidate the vaccine’s efficacy in diverse breast cancer populations.

In light of these findings and limitations, the next research phase should prioritize refining patient stratification criteria. Investigating the potential synergies of combining AE37 with other therapeutic modalities and exploring its use in neoadjuvant or adjuvant settings could further enhance its clinical utility. Long-term follow-up studies assessing the durability of immune responses and their correlation with clinical outcomes are crucial for establishing the vaccine’s sustained efficacy. Moreover, leveraging advanced immunogenomic techniques to profile individual patient immune responses may unlock critical insights into predicting vaccine responsiveness. Future studies could benefit from integrating translational research approaches to bridge the gap between bench and bedside, facilitating a more comprehensive understanding of the vaccine’s mechanisms of action.

## 5. Limitations of study

While the AE37 HER2-targeted vaccine studies provide valuable insights, it is essential to acknowledge certain limitations that temper the interpretation of their findings. The studies focus on the AE37 vaccine in isolation, overlooking potential synergies with other therapeutic modalities. Future investigations could explore combination therapies to exploit complementary mechanisms and enhance treatment outcomes. Also, the exclusion of non-English language papers introduces language bias. Restricting the review to English-language publications, there is a risk of overlooking valuable insights, data, or perspectives present in studies conducted in other languages. This bias may limit the global applicability of the findings.

## 6. Conclusion

The AE37 HER2-targeted vaccine emerges as a promising contender in breast cancer immunotherapy, displaying a favorable safety profile and demonstrating potential efficacy in specific patient subgroups. The safety outcomes consistently revealed minimal adverse effects across the studies, affirming the tolerability of the AE37 vaccine. This is a crucial foundation for its further exploration and potential integration into breast cancer treatment paradigms. Efficacy outcomes, while variable, highlight the importance of precise patient stratification. Subgroup analyses suggest potential benefits in advanced-stage, HER2 under-expressed, and triple-negative breast cancer patients. Forward, the implications of these findings call for refined patient stratification, considering factors like HER2 expression and disease stage. The identified limitations underscore the need for continued research, exploring combination therapies, refining dosing strategies, and leveraging advanced immunogenomic techniques for personalized treatment.

## Author contributions

**Conceptualization:** Nicholas Aderinto.

**Writing – original draft:** Gbolahan Olatunji, Emmanuel Kokori, Ismaila Ajayi Yusuf, Olumide Akinmoju, Timilehin Isarinade, Rosemary Komolafe, Aminat Akinoso, Adeola Akinboade, Osadebamwen Osaghae, Muili Abdulbasit, Nicholas Aderinto.

**Writing – review & editing:** Gbolahan Olatunji, Emmanuel Kokori, Ismaila Ajayi Yusuf, Olumide Akinmoju, Timilehin Isarinade, Rosemary Komolafe, Aminat Akinoso, Adeola Akinboade, Osadebamwen Osaghae, Muili Abdulbasit, Nicholas Aderinto.

## References

[1] Łukasiewicz S Czeczelewski M Forma A. Breast cancer-epidemiology, risk factors, classification, prognostic markers, and current treatment strategies-an updated review. Cancers (Basel). 2021;13:4287. 34503097 10.3390/cancers13174287 PMC8428369

[2] Courtney D Davey MG Moloney BM. Breast cancer recurrence: factors impacting occurrence and survival. Ir J Med Sci. 2022;191:2501–10. 35076871 10.1007/s11845-022-02926-x PMC9671998

[3] Lafourcade A His M Baglietto L. Factors associated with breast cancer recurrences or mortality and dynamic prediction of death using history of cancer recurrences: the French E3N cohort. BMC Cancer. 2018;18:171. 29426294 10.1186/s12885-018-4076-4 PMC5807734

[4] Ahmad A. Pathways to breast cancer recurrence. ISRN Oncol. 2013;2013:290568. 23533807 10.1155/2013/290568 PMC3603357

[5] Pallerla S Abdul AURM Comeau J. Cancer vaccines, treatment of the future: with emphasis on HER2-positive breast cancer. Int J Mol Sci. 2021;22:779. 33466691 10.3390/ijms22020779 PMC7828795

[6] Cronin KA Harlan LC Dodd KW. Population-based estimate of the prevalence of HER-2 positive breast cancer tumors for early stage patients in the US. Cancer Invest. 2010;28:963–8. 20690807 10.3109/07357907.2010.496759 PMC5094051

[7] Yang J Ju J Guo L. Prediction of HER2-positive breast cancer recurrence and metastasis risk from histopathological images and clinical information via multimodal deep learning. Comput Struct Biotechnol J. 2021;20:333–42. 35035786 10.1016/j.csbj.2021.12.028 PMC8733169

[8] You Z Zhou W Weng J. Application of HER2 peptide vaccines in patients with breast cancer: a systematic review and meta-analysis. Cancer Cell Int. 2021;21:489. 34526020 10.1186/s12935-021-02187-1 PMC8442296

[9] Mittendorf EA Ardavanis A Symanowski J. Primary analysis of a prospective, randomised, single-blinded phase II trial evaluating the HER2 peptide AE37 vaccine in breast cancer patients to prevent recurrence. Ann Oncol. 2016;27:1241–8. 27029708 10.1093/annonc/mdw150 PMC4922316

[10] Sears AK Perez SA Clifton GT. AE37: a novel T-cell-eliciting vaccine for breast cancer. Expert Opin Biol Ther. 2011;11:1543–50. 21895539 10.1517/14712598.2011.616889

[11] Brown TA 2nd Mittendorf EA Hale DF. Prospective, randomised, single-blinded, multicenter phase II trial of two HER2 peptide vaccines, GP2 and AE37, in breast cancer patients to prevent recurrence. Breast Cancer Res Treat. 2020;181:391–401. 32323103 10.1007/s10549-020-05638-x PMC7188712

[12] Peace KM Mittendorf EA Perez SA. Subgroup efficacy evaluation of the AE37 HER2 vaccine in breast cancer patients in the adjuvant setting. J Clin Oncol. 2017;35(15_suppl):3088–3088.

[13] AE37. Immuno-Oncology News. Available at: https://immuno-oncologynews.com/ae37/ [access date October 3, 2023].

[14] Tobias J Garner-Spitzer E Drinić M. Vaccination against Her-2/neu, with focus on peptide-based vaccines. ESMO Open. 2022;7:100361. 35026721 10.1016/j.esmoop.2021.100361 PMC8760406

[15] Anastasopoulou EA Voutsas IF Papamichail M. MHC class II tetramer analyses in AE37-vaccinated prostate cancer patients reveal vaccine-specific polyfunctional and long-lasting CD4(+) T-cells. Oncoimmunology. 2016;5:e1178439. Published May 2, 2016. 27622033 10.1080/2162402X.2016.1178439 PMC5006925

[16] Gates JD Clifton GT Benavides LC. Circulating regulatory T cells (CD4+CD25+FOXP3+) decrease in breast cancer patients after vaccination with a modified MHC class II HER2/neu (AE37) peptide. Vaccine. 2010;28:7476–82. 20858449 10.1016/j.vaccine.2010.09.029

[17] Schneble EJ Berry JS Trappey AF. Vaccine-specific T-cell proliferation in response to a dual peptide cancer vaccine in breast and ovarian cancer patients. J Immunother Cancer. 2013;1(Suppl 1):P236.

